# Rib fracture after stereotactic radiotherapy on follow-up thin-section computed tomography in 177 primary lung cancer patients

**DOI:** 10.1186/1748-717X-6-137

**Published:** 2011-10-13

**Authors:** Atsushi Nambu, Hiroshi Onishi, Shinichi Aoki, Tsuyota Koshiishi, Kengo Kuriyama, Takafumi Komiyama, Kan Marino, Masayuki Araya, Ryo Saito, Lichto Tominaga, Yoshiyasu Maehata, Eiichi Sawada, Tsutomu Araki

**Affiliations:** 1 Department of Radiology, University of Yamanashi, Chuo City, Japan; 2 Department of Radiology, Kofu Municipal Hospital, Kofu City, Japan; 3 Department of Radiology, Yamanashi Prefectural Central Hospital, Kofu City, Japan

**Keywords:** stereotactic radiotherapy, lung cancer, rib fracture, thin-section CT

## Abstract

**Background:**

Chest wall injury after stereotactic radiotherapy (SRT) for primary lung cancer has recently been reported. However, its detailed imaging findings are not clarified. So this study aimed to fully characterize the findings on computed tomography (CT), appearance time and frequency of chest wall injury after stereotactic radiotherapy (SRT) for primary lung cancer

**Materials and methods:**

A total of 177 patients who had undergone SRT were prospectively evaluated for periodical follow-up thin-section CT with special attention to chest wall injury. The time at which CT findings of chest wall injury appeared was assessed. Related clinical symptoms were also evaluated.

**Results:**

Rib fracture was identified on follow-up CT in 41 patients (23.2%). Rib fractures appeared at a mean of 21.2 months after the completion of SRT (range, 4 -58 months). Chest wall edema, thinning of the cortex and osteosclerosis were findings frequently associated with, and tending to precede rib fractures. No patients with rib fracture showed tumors > 16 mm from the adjacent chest wall. Chest wall pain was seen in 18 of 177 patients (10.2%), of whom 14 patients developed rib fracture. No patients complained of Grade 3 or more symptoms.

**Conclusion:**

Rib fracture is frequently seen after SRT for lung cancer on CT, and is often associated with chest wall edema, thinning of the cortex and osteosclerosis. However, related chest wall pain is less frequent and is generally mild if present.

## Background

Stereotactic radiotherapy (SRT) for primary lung cancer has recently attracted attention because of its promising treatment effects [1-10]. A recent report demonstrated that SRT achieved a good survival rate for patients with non-small cell lung carcinoma, comparable to those of surgery [10]. SRT has now been applied not only to medically inoperable patients but also to operable ones. In the near future, SRT might become an alternative treatment to surgery for stage I non-small lung carcinoma.

One major concern that must always been taken into consideration when selecting treatment methods is treatment sequelae. SRT is generally considered a safe treatment, with fewer complications than surgery. However, several studies have reported complications in SRT, such as radiation pneumonitis [11,12] and chest wall injury, including rib fracture [5-7,13-16]. Frequencies of rib fracture after SRT have already been reported in several investigations. However, detailed CT findings of chest wall injury have yet to be clarified.

The present study therefore aimed to fully characterize detailed CT findings of chest wall injury after SRT for primary lung cancer using thin-section CT.

## Methods

The institutional review board approved all study protocols. Written informed consent was obtained from all patients prior to participation in this study.

### Patients

Between November 2001 and April 2009, a total of 210 patients with primary non-small cell lung carcinoma underwent SRT in our institution. Of these patients, 177 patients agreed to participate in this prospective study. Patient characteristics are summarized in Table 1.

**Table 1:** Characteristics of the 177 primary lung cancer patients enrolled in this study.

	Lung cancer patients (n = 177)
*Average age(range)	77.3 ± 7.0 (55-92)

*Gender (male: female)	132:45

**Range of follow-up period (median)	11-99 (27)

Tumor diameter (average ± standard deviation)	8-55 mm(30.0 ± 9.1)

central tumors: peripheral tumors	22:155

Method of radiotherapy (48Gy/4fr:60Gr/10fr:70Gr/10fr)	75:37:65

*Presented as mean ± standard deviation.

**Presented as median.

### Methods of radiotherapy

SRT was performed using noncoplanar 10 dynamic arcs. A total dose of 48-70Gy at the isocenter was administered in 4-10 fractions, and approximately 80% isodose line of prescribed dose covered planning target volume (PTV) using a 6 MV X-ray, comprising three different methods, namely 48Gy/4fractions, 60Gy/10fractions, and 70Gy/10fractions, (Table 1). We essentially used 60Gy/10fractions but when tumor measured more than 3 cm (i.e. T2) 70Gy/10fractions was used, and cases that were registered in a certain clinical trial were treated with 48Gy/4fractions. The dose was not constrained by surrounding normal tissues including chest wall. Heterogeneity corrections were made in all cases.

After adjusting the isocenter of the PTV to the planned position in a unit comprising a CT scanner and linear accelerator, irradiation was performed under patient-controlled breath-holding and radiation beam switching.

### CT examination

Preradiotherapeutic and follow-up CT were performed using the same 16 multidetector row scanner (Aquilion 16 (Toshiba Medical Systems, Otawara, Japan)) and with the identical protocols.

Parameters for CT scanning were as follows: peak voltage 120 kVp, tube rotation time 0.5 second, slice collimation 1.0 mm, and beam pitch 0.94. Tube currents were determined by an automatic exposure control with the noise factor for determining the applied tube current was set at 11 (standard deviation) and the tube currents actually ranging from 110 to 400 mA.

Contrast-enhanced CT was performed for 116 patients (67.1%) after unenhanced CT. Contrast material (Omnipaque 300, Daiichi Sankyo, Tokyo) in a volume tailored to the body weight of each patient (600 mg iodine/kg body weight) was injected from the anterior cubital vein within a fixed injection time of 50 s (i.e. injection rate was variable.). CT scans were started at 60 and 120 s after beginning of the contrast injection.

These data were reconstructed into 5 mm sections. Thin-section CT (slice thickness, 1 mm) was also produced for regions that included tumor or radiation-induced opacities targeting the affected lung, which was mainly used for the evaluation of chest wall injury.

Preradiotherapeutic CT was performed within 1 month before SRT, while follow-up CT was performed at 3 and 6 months after the completion of the radiotherapy, and every 6 months thereafter.

### CT evaluation

Preradiotherapeutic CT was interpreted by either of two chest radiologists (A.N, E.S) in our institution. Maximum tumor size and the shortest distance between the tumor margin and chest wall (tumor-chest wall distance) were measured on 1 mm contrast-enhanced CT with a reconstruction kernel for viewing lung parenchyma as a part of the radiology report. Maximum tumor size was defined as the maximum dimension of a tumor in all of axial CT sections that included the tumor.

Follow-up CT was also examined by either of the same radiologists with special attention to abnormal findings of the chest wall in addition to routine radiological assessment. Rib fracture in this study was defined as a disruption of cortical continuity with malalignment. Thinning of cortex was defined as a focal area of cortex with a thickness less than half of the surrounding normal cortex. Osteosclerosis was defined as an area of increased attenuation comparable to cortex in the medulla of rib.

The time at which each finding first appeared after the completion of SRT was reviewed. Final outcomes of rib fractures during the follow-up period were also assessed on follow-up CT.

### Follow-up of patients

Every patient was basically asked to visit our clinic at 3, 6, and every 6 months thereafter after the completion of radiotherapy. At every visit, a thorough examination was performed, consisting of inquiry focusing on pain at the chest wall near the irradiated tumor and respiratory symptoms, physical examination by an attending radiation oncologist, blood test, and CT. Clinical symptoms considered related to chest wall injury after SRT were graded according to the criteria for pain in Common Terminology Criteria for Adverse Events, *version. 3*. Chest radiologists interpreted the results of CT just after the examinations. If the patient complained of pain, analgesics were prescribed as appropriate.

### Evaluation of dosimetry

**Figure 1:**
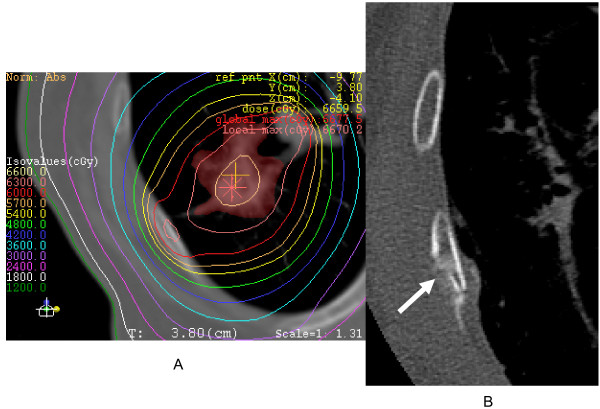
**An 86-year old woman with adenocarcinoma**. A:Dosimetry overlaying CT shows the maximum prescribed dose of chest wall as 63Gy, with a BED_3 _of 233.2Gy. B: Rib fracture was noted at 24 months after completion of SRT. Amorphous osteosclerosis is also seen (arrow).

Among the 177 patients, detailed dosimetries were available for review in 26 patients with rib fracture and 22 patients without. Patients without fracture were randomly sampled among those with no evidence of fracture on CT for more than 30 months. We set this period as a cut-off point as most rib fractures after SRT in this series had occurred within 30 months after completion of SRT. At the point on the chest wall that had received the maximum dose, BED was calculated in each case assuming the α/β ratio as 3 (BED_3_) (Figure 1). The chest wall volume (cc) that received in BED_3 _≥ 50 Gy was also calculated.

### Data analysis

Data analyses were performed retrospectively using the prospectively interpreted radiology reports.

First, we calculated the crude incidence of rib fracture after SRT on follow-up CT during the follow-up periods of the patients. As crude incidence may underestimate actual incidence of rib fracture, we also performed a Kaplan-Meier method to obtain a more accurate estimate of incidence of rib fracture. We also assessed the relationship between rib fracture and related findings in terms of time frame.

Second, we determined the threshold tumor-chest wall distance on preradiotherapeutic CT to discriminate patients who with rib fractures from those without. Frequencies of rib fracture when the tumor-chest wall distance was less than or equal to the threshold distance and when the distance was 0 mm were also calculated.

Third, we evaluated the frequency of clinical symptoms.

Fourth, mean BED_3 _and BED_3 _≥ 50 Gy were calculated in fracture and non-fracture groups and were compared between the two groups using unpaired t test. Fisher's exact test or χ^2 ^test was used to see differences between groups.

Value of *p *< 0.05 were considered statistically significant.

All statistical analyses were performed using IBM SPSS Statistics version 18(New York, USA).

## Results

### Frequency of rib fractures after SRT

The crude incidence of rib fracture was 23.2% (41/177) at a median follow-up of 27 months (Table 2). The frequency of rib fracture was not statistically different among the three different dose fractionations (χ2 test, p = 0.391). Kaplan-Meier method estimated the incidence to be 27.4% at 24 months.

**Table 2:** Appearance time and frequencies of the rib fractures and related findings

	Appearance time ranges (months)*	Crude frequency of each finding	Frequency at 24 months by Kaplan-Meier method
Rib fracture	21.2 (4-58)	41/177(23.2%)	27.4%

Thinning of the cortex	15.6 (4-36)	36/177 (20.3%)	

Osteosclerosis	14.7 (4-57)	26/177(14.7%)	

Chest wall edema	12.0 (2-57)	45/177 (25.4%)	

*Presented as mean (range).

### Imaging findings of rib fracture and related findings and appearance times

**Figure 2:**
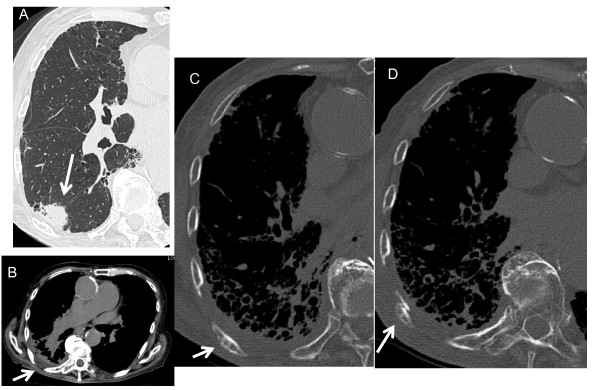
**An 85-year-old man with a rib fracture after SRT**. A, A preradiotherapeutic thin-section CT showing a spiculated nodule with air-containing spaces (arrow). B, Seven months later after SRT, CT shows edema of the right chest wall adjacent to the tumor, as evidenced by asymmetrical swelling and effacement of the fat planes (arrow). C, On follow-up CT at 13 months after SRT, thin-section CT with a bone window setting demonstrates thinning of cortex with mild sclerotic foci of the medulla in a rib. D, At 20 months after SRT, rib fracture with malalignment of the cortex is apparent (arrow).

**Figure 3:**
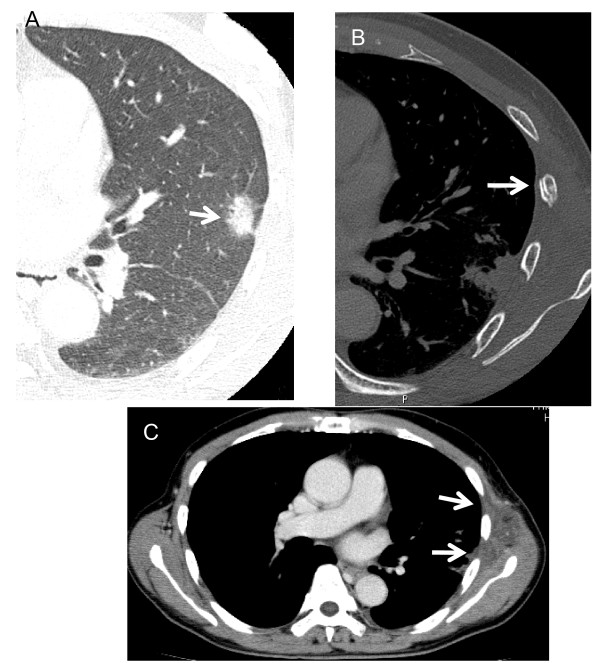
**A 65-year-old man with a rib fracture after SRT**. A, Preradiotherapeutic thin-section CT showing a spiculated nodule with surrounding ground-glass opacity close to the chest wall (arrow).  B, Twelve months later after SRT, a rib fracture is apparent (arrow). C, At 6 months after SRT, enhanced CT shows swelling of the left chest wall with an area of low attenuation (arrows).

**Figure 4:**
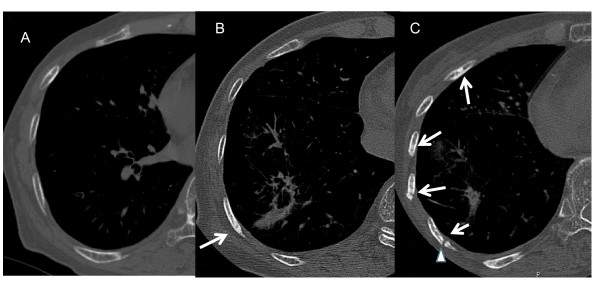
**A 85-year-old woman with adenocarcinoma**. A, Preradiotherapeutic thin-section CT at the bone window shows no marked abnormality of the ribs. B, At 18 months after SRT, bone sclerosis of the rib adjacent to the lung tumor appeared (arrow). C, At 30 months after completion of SRT, multiple rib fractures with areas of sclerosis are seen. Pseudoarthrosis is present in one of the fractured bones (arrow head).

Results of appearance time and frequency of rib fractures are summarized in Table 2. Rib fractures appeared at a mean of 21.2 months (range, 4 -58 months) on follow-up CT. Fractures invariably occurred at the ribs close to the irradiated tumor, and were solitary or multiple (Figure 2). Final outcomes for fractures were non-union in 28 patients, including 14 patients with pseudoarthrosis (defined as covering of cortex over the fractured surface), and bony union in 13. Chest wall edema was seen in 45 of 177 patients (25.4%), appearing at a mean of 12 months after SRT (range, 2 -57 months). Such edema was seen as asymmetrical swelling of the ipsilateral chest wall compared with the contralateral chest wall along with effacement of interlaced intramuscular fat attenuation. Low-attenuation areas in the chest wall were occasionally associated, which became more conspicuous on contrast- enhanced CT (Figure 3). Thinning of the cortex was observed in 36 patients (30.3%) at 4 to 36 months. Osteosclerosis was evident in 26 patients (14.7%) on follow-up CT at a mean of 15 months (range, 4-57 months). This finding appeared as mottled sclerosis of the affected bone (Figure 4). These findings related to rib fracture typically preceded the identification of rib fracture.

### Symptoms of rib fracture

Clinical symptoms in patients with rib fracture and without rib fracture are summarized in Table 3. Chest wall pain was seen in 18 of 177 patients (10.2%), of whom 14 patients developed rib fracture. No patients complained of Grade 3 or more symptoms. Four patients without rib fractures complained of Grade 1 chest wall pain with all 4 cases showing radiological evidence of chest wall edema. In the study population as a whole, the frequency of chest wall pain was 21.5% (38/177). The frequency of chest wall pain was not significantly different between the patients with union (6/13, 46%) and non-union (7/28, 25%) rib fracture (Fisher's exact test, p = 0.160).

**Table 3:** Frequency and degree of chest wall pain

Degree of pain*	Fracture group (n = 41)	Non-fracture group (n = 136)
Grade 0	27 (65.9)	132(97)

Grade 1	7 (17.1)	4(3)

Grade 2	7 (17.1)	0(0)

Grade 3 and 4	0 (0)	0(0)

*The degree of chest wall pain was evaluated according to Common Terminology Criteria for Adverse Events, *Ver. 3*.

**The numbers in the parentheses are percentages

### Threshold tumor-chest wall distance in the occurrence of rib fracture

Mean tumor-chest wall distance was 12.3 mm (range, 0 - 53 mm). No patients with rib fracture showed a tumor-chest wall distance > 16 mm, while frequency of rib fracture was 31.3% (41/131) for a distance ≤ 16 mm, and 37.1% at 24 months by Kaplan-Meier method. When the distance was 0 mm, frequency of rib fracture was 36.7% (22/60) and 51.8% at 24 months by Kaplan-Meier method (Table 4).

**Table 4:** Frequency of rib fracture in relation to tumor-chest wall distance

Tumor-chest wall distance(mm)	Crude frequency	Frequency at 24 months by Kaplan-Meier method
≤25	41/148(27.8%)	33.2%

≤16	30/131(31.3%)	37.1%

0	22/60(36.7%)	51.8%

*The numbers in the parentheses in frequency are percentages.

Maximum BED3 of the chest wall in patients with and without rib fracture, and threshold dose for rib fracture occurrence Mean BED3 of the chest wall was 240.7 ± 38.7 in 26 patients with rib fracture and 146.8 ± 74.5 in 22 patients without rib fracture, representing a significant difference between groups (p < 0.001). The lowest BED3 that resulted in rib fracture was 152.4 Gy. Mean chest wall volume (cc) with BED3 > 50Gy was 110.3 ± 45.0cc in the fracture group and 50.1 ± 59.8 in the non- fracture group, again representing a significant difference (p < 0.001). The minimum volume that resulted in rib fracture was 25cc.

## Discussion

Our results demonstrated that the development of rib fracture after SRT is not uncommon with a frequency of 23.2% for the whole study population. Not unexpectedly, frequency increased with closer proximity of the tumors to the chest wall, from 31.3% ≤16 mm to 36.7% at 0 mm. The reported frequencies of rib fracture after SRT vary widely among investigators, ranging from 3% to 21.2% [5-7,13-16]. Our result is closest to that reported by Petterson, et al., who reported the highest frequency (21.2%) among the previous reports [14]. We speculate that these discrepancies are mainly caused by differences in the methods for estimating frequency. Petterson, et al. and the present study obtained frequencies based on follow-up CT, whereas other studies based frequencies on findings for patients who complained symptoms. That is, differences may be largely due to whether asymptomatic patients with rib fracture were likely to be included in frequency calculations. Our clinical experience supports this speculation. Differences in follow-up periods, methods of SRT or the proportion of tumors close to the chest wall may also have contributed to the discrepancies between studies. The frequency of rib fracture reported by Petterson, et al. is still lower than our result despite the fact that they used a higher prescribed SRT dose than we did. This may be because thin-section CT in the present study may have allowed sensitive detection of rib fracture.

In Kaplan-Meier method, the frequency of rib fracture was calculated to be even higher (27.4% at 24 months). This incidence is considered to be a more accurate estimate of frequency of rib fracture as there were censored cases during the follow-up periods.

The frequency of rib fracture is also more common in SRT for lung cancer than in breast conserving surgery combined with radiotherapy, which has a reported frequency of 0.3-2.2% [17,18], probably due to much higher dose delivered to the rib in SRT when tumors are close to the chest wall.

Rib fractures occurred at a mean of 21.2 months (range, 4-58 months) after SRT, mostly within 30 months after completion of SRT, and were frequently preceded by chest wall edema, thinning of the cortex of the rib or sclerosis of the medulla of the rib. We may summarize the typical course of chest wall injury after SRT as depicted on thin-section CT as follows: at several months after SRT chest wall edema first appears. The cortex then becomes thinner and the medulla sometimes becomes sclerotic in a mottled fashion, and the affected rib eventually undergoes fracture. These CT findings presumably correspond to soft tissue edema and changes in bone vascularity due to increased permeability or occlusion of the capillaries caused by irradiation of the soft tissue, and a decrease in number of osteoblasts resulting in decreased collagen production, in turn causing osteopenia and subsequent bone injury [19]. Osteosclerosis after radiotherapy is considered to represent reactive bone formation caused by remaining osteoblast cells [20].

Under such conditions, the rib becomes extremely vulnerable and often fractures. Although these bone changes may actually represent insufficiency fracture [19], radiation osteitis [21], callous formation secondary to microtrabecular fracture or osteonecrosis [22], we did not use these terms as we had no pathological confirmation of such findings. We therefore employed the common terms for imaging findings.

We think that these preceding findings may be usable as predictors of rib fracture. Prediction of rib fracture may be informative to the referring physicians as well as to patients as we might initiate treatment for chest wall pain related to the forthcoming rib fracture in advance or possibly take some preventive measures against rib fractures. Although the frequency of clinical symptoms was not high in patient with rib fracture and the clinical symptoms were generally not severe, most symptomatic patients had rib fracture. Therefore, prediction of rib fracture will clinically be important.

In addition, bone sclerosis or focal loss of cortex may be mistaken for metastasis. Familiarity with these findings will therefore minimize the potential for confusion.

The outcomes of rib fracture were non-union in 28 patients, including 14 patients with pseudoarthrosis and bony union in 13. Needless to say, the proportion of union and non-union largely depends on the duration of follow-up and the prescribed dose to tumors. However, we can at least say that a substantial proportion of rib fractures after SRT for lung cancer can remain a state of non-union for a long time after SRT and that pseudoarthrosis is not uncommon. However, the outcomes of rib fracture seem unrelated to the frequency of clinical symptoms.

A tumor-chest wall distance of 16 mm appears to represent a threshold value, beyond which rib fracture did not occur, in our series. This threshold offers a concise and convenient reference value. Undoubtedly, the risk of rib fractures depends much more on the dose delivered to the rib and therefore a dosimetry-based evaluation can provide a more accurate estimate of the risk of rib fractures. However, dosimetry can only be produced after SRT is chosen as the treatment. Our approach can provide a patient or referring physician with information about the risk of rib fracture based only on preradiotherapeutic CT before decision is made to undergo SRT. Our result may not be simply applicable to patients in other institutions as prescribed doses differ among institutions, but will be valid when prescribed doses are less than or equal to our own.

Mean BED_3 _of the chest wall (240.7 ± 38.7 Gy) and mean chest wall volume (cc) with BED_3 _≥ 50Gy (110.3 ± 45.0cc) in 26 patients with rib fracture were much higher than those (146.8 ± 74.5Gy and 50.1 ± 59.8cc) in 22 patients without rib fracture, with statistical significances, respectively. These values may also be usable to predict the risk of rib fracture. The lowest BED_3 _that resulted in rib fracture was 152.4Gy. The threshold BED_3 _for producing rib fracture seemed to be around 150Gy, but further investigation is necessary to make a definitive conclusion.

This study has some limitations that must be considered. First, we regarded the appearance time of rib fracture and other related findings as that when these findings were first seen on follow-up CT. However, these events would actually have occurred within the interval of time since the previous CT. The present study would thus have overestimated time that elapsed until these events.

Second, for BED_3 _of chest wall, only a small number of cases from the study population were sampled. This was because of the limited capability of our treatment planning computer for data handling, which requires a substantial amount of time to reproduce a dosimetry. Calculating dosimetries of all cases is obviously the best way to obtain a threshold BED, but we believe that our random sampling method provided a clear and concise reference value, which would offer a benchmark when considering risk of rib fracture in clinical practice. Third, the method of SRT for lung cancer has yet to be standardized. So, our results cannot be simply applied to other institutions.

## Conclusion

Rib fracture is seen with high frequency after SRT for lung cancer when the tumor is close to the chest wall. Chest wall edema and thinning and osteosclerosis of the cortex represent related findings that often precede rib fracture and might be predictive of a forthcoming rib fracture. However, related chest wall pain is less frequent and is generally mild if present.

## Competing interests

The authors declare that they have no competing interests.

## Authors' contributions

All authors approved read and approved the final version of this paper. AN is the first author of this paper involved in interpretation of CT, clinical data collection, statistical analysis and drafting this paper. HO carried out clinical data collection, supervision of this study, editing and approving the paper. SA carried out clinical data collection, dosimetry calculation and revision of clinical data. TK, ES and LT carried out collection of CT data and clinical data. KK, TK, KM, MA, RS and YM carried out clinical evaluations of patient at follow-up visits. TA carried out supervision of this study and final approval of this paper.
